# Determinants of Nursing Process Implementation in North East Ethiopia: Cross-Sectional Study

**DOI:** 10.1155/2018/7940854

**Published:** 2018-09-06

**Authors:** Yihun Miskir, Solomon Emishaw

**Affiliations:** Department of Nursing, College of Medicine and Health Science, Bahir Dar University, Bahir Dar, Ethiopia

## Abstract

Nursing process is a framework used to provide an effective, coordinated, and organized quality care for patients. Effective implementation of this framework leads to improved quality of care and decreases potential complication, hospital length of stay, and the cost of care. To assess implementation of nursing process and its hindering factors, a quantitative cross-sectional study was conducted among nurses in Afar region hospitals from October 2016 to December 2016. The data were collected from 102 nurses using primary Brooking's ward nurses' self-report questionnaire and with some newly prepared questions. The collected data were entered using Epi-Data version 3.1 and analyzed by SPSS version 20 and then presented by tables, graphs, and figures. Forty-three (42.1%) nurses were implementing nursing process at the time of data collection. Assessment and diagnosis were carried out by 57 (56.9%) nurses, planning by 46% of nurses, implementation by 38.2% of nurses, and evaluation by 36.2% of nurses in Afar region. Among the hindering factors towards nursing process implementation, lack of preparedness or knowledge about the nursing process or some part of it (83.3%) and absence of in-service training pertinent to nursing process (75.5%) were the most mentioned ones. Generally, nursing process was poorly implemented in Afar region mainly due to lack of knowledge and absence of in service training. Therefore, giving emphasis for cognitive parts of students about nursing process during their school time and refreshing nurse staffs with continuous training will definitively improve level of nursing process implementation.

## 1. Introduction

It is considered that contemporary nursing has been shaped and recognized as an independent profession since the day of Florence Nightingale. Being recognized as a profession, the discipline has started to establish its own conceptual and theoretical framework and, of them, nursing process is the peculiar one [1].

Some studies indicate that the origin of nursing process is traced back to 1955 when Hall a nursing theorist described nursing care as a process [2, 3]. Other authors believed that the term was developed and operationalized during the 1960s by Yura and Walsh [4].

Whatever the time of its origin and whoever the nominator, the process currently is a highly recommended standard of care, widely accepted, and used in scientific method to guide procedures and qualify nursing care [5, 6]. More recently, the process is being defined as a systematic, organized, and dynamic way used by nurses to meet the individualized healthcare needs of their patients through operating five interrelated steps: assessment, diagnosis, planning, implementation, and evaluation [3, 7, 8].

Effective implementation of nursing process leads to improved quality of care, facilitates, and healing process and in doing so minimizes hospital stay, increases patient satisfaction [5], and increases service utilization [9]. As patient's hospital stay decreases, the cost of the healthcare system decreases and patient's working time increases. Therefore, it also has economical aspect.

Furthermore, nursing process enables nurses to perform their activities with logical justification and helps them to function as an autonomous and distinct profession [2, 9, 10]. Studies claim that, by implementing nursing process, nursing profession will be strengthened, internationalized, and dignified and patient care criteria will be unified [11]. Orlando also argues that if an activity is provided for a client without assessing his need and if that action brings change, then nursing is not a profession [9]. According to her assumption, for nursing to be a profession and to be recognized as an independent, nurses should follow stepwise activities that the preceding activities determine the succeeding one, which is nursing process.

In spite of the aforementioned importance of implementing nursing process, a significant number of nurses do not apply it in practice [5, 7, 11–13]. Lack of knowledge about the process, inadequate training, patient to nurse ratio, resource scarcity, low salary and poor promotion of nurses, influence from other health care members, etc. are some of the hindering factors identified by previous studies [2, 5, 12–16].

In Afar region however, nursing process implementation and the hindering factors were not known and, as to our knowledge, there was no up to date study. It was, therefore, compulsory to conduct this research with the aim of identifying the degree of nursing process implementation and possible hindering factors.

## 2. Methods and Materials

### 2.1. Study Area and Period

The study was conducted from October to December 2016 in Afar regional state, one of the 9 regions in Ethiopia. Afar regional state has a population size of over 2 million. Its average elevation is 400 m above sea level and hence has unfavorable climate and hot weather condition. There were 6 governmental hospitals staffed by 130 nurses. These include Muhamed Akilel, Dubti, Asayita, Dalifagee, Kelewa, and Abala hospital. Among these Kelewa and Abala were the newly established hospitals and staffed only by 8 nurses each.

### 2.2. Study Design

Institution-based quantitative cross-sectional study was used.

### 2.3. Source Population and Study Participants

All nurses working in the hospitals were used as a source population. Selected nurses working in the hospitals participated in the study.

### 2.4. Inclusion Criteria

Nurses available during the data collection period were included in the study.

### 2.5. Exclusion Criteria

Free service worker nurses were excluded.

### 2.6. Sample Size and Sampling Procedure

The sample size was determined using single population proportion formula by considering normal distribution (Z=1.96) with confidence interval of 95% and population proportion of nurses implementing nursing process in Afar region 50% as there were no previous studies conducted in the region. Using correction formula the final sample size becomes 97 and by adding 10% of nonresponse rate, 107 nurses were selected for the study.

The number of nurses from each hospital was obtained by proportionally allocating the final sample size and then simple random sampling technique was employed to select the allocated number of nurses from each hospital.

### 2.7. Data Collection

Data regarding implementation of nursing process were collected using self-administered questionnaire [4] and data regarding hindering factors were collected by using newly prepared and pre-tested self-administered questionnaires.

### 2.8. Data Analysis

Data were cleaned, coded, entered to Epi-data version 3.1, and analyzed by SPSS version 20.

### 2.9. Operational Definitions

**Implementation of nursing process** in the five Likert scale is never, sometimes, I do not know, usually, and always; those responses of usually and always were taken as implemented and the other responses as not implemented.

### 2.10. Study Variables

#### 2.10.1. Dependent Variable

(i) Implementation of nursing process.

#### 2.10.2. Independent Variable

- Sociodemographic variables
  - sex, age, monthly income, and level of education
- Other variables
  - institution from where educational award is obtained, nurse to patient ratio, working hours per day, year of experience, hospital unit, recent training or workshop participation, and adequacy of time allotted for nursing process at pre service training.

### 2.11. Ethical Considerations

Before the data collection, ethical clearance letter was obtained from ethical review committee of Samara university research and community service core process. The respondents were informed about the purpose of the study, and their oral consent was obtained. The respondents' right to refuse or withdraw from participating in the study was fully maintained and the information provided by each respondent is kept strictly confidential.

## 3. Result

Out of 107 questionnaires distributed among the study participants, 102 were filled completely and returned to making the response rate 95.3%.

### 3.1. Sociodemographic Characteristics of the Respondents

From 102 respondents 54 (52.9%) were males. Majority 55 (53.9%) of respondents were under the age group of 25-29. Sixty-seven (65.7%) respondents had diploma in educational status and the majority of respondents 76 (74.5%) obtained their awards from governmental institution. Regarding experience, most (52.9%) of respondents were not experienced as they had 4 and below 4 years of experience. More than half of the respondents 60 (58.8%) were working 8 or below 8 hours per day during the data collection time and 44 (43.1%) of them were serving 5-10 patients at a time (Table 1).

### 3.2. Nursing Process Implementation

#### 3.2.1. Assessment and Diagnosis

When a new patient was admitted to the ward, most nurses 60 (58.5%) agreed that an initial patient assessment was always carried out before planning and 41 (40.2%) of nurses usually use a specific form to conduct assessment while 33 (32.4%) nurses always use a specific form. Exactly half, 51 (50%), of the nurses responded that the assessment, if conducted, is always conducted within the first 24 hours of patient admission. Most nurses, 73 (71.5%), indicated that they identified and documented the nursing problems of the patient. About 57 (55.9%) of the nurses reported that patients'/or patient relatives' opinions were systematically taken in to account (Table 2).

#### 3.2.2. Nursing Plan

Taking “usually” and “always” responses as “implemented,” sixty-seven (65.7%) of respondents reported that they prepared a written care plan that incorporates the nursing problem and goals identified for resolution of those problems before carrying out nursing interventions. The majority of the respondents 73 (71.6%) reported that nursing interventions were identified and documented in care plan even though they were not always with enough detail (Table 2).

#### 3.2.3. Implementation

Sixty (58.8%) nurses have said that they reassessed patient's condition before implementing any planned nursing intervention in order to be sure of its appropriateness. Most of the respondents, 71 (69.6%), reported that they take part in medical round for their patients. But more than half of the respondents have not been allocated to the same patients for several days (Table 2).

#### 3.2.4. Evaluation

About 57 (55.9%) nurses systematically evaluated the effectiveness of the care and 56 (54.9%) of nurses recorded the evaluation in the care plan. But more than half of the nurses 58 (56.9%) did not include patients/relatives in the evaluation of care on the ward (Table 2).

### 3.3. Summary of Nursing Process Implementation

**Figure 1:**
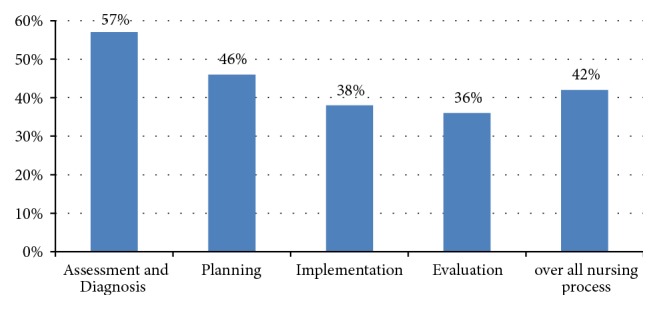
Nursing process implementation by its parts and overall as a process among nurses in Afar region hospitals, Afar, north East Ethiopia, 2016 (n= 102).

The overall computed result shows that “assessment and diagnosis” part of the nursing process as performed by more than half 57 (56.9%) of nurses followed by planning 47 (46%) whereas implementation and evaluation parts were the least applied ones in the study areas. Generally, the degree of implementation of nursing process was 43 (42.1%) (see Figure 1).

### 3.4. Binary Logistic Analysis of Nursing Process Implementation with Selected Variables

From binary logistic regression analysis shown in Table 3 male nurses were 2 times and BSC nurses, nurses in the age group ≥30, and nurses graduated from governmental institutions were 4 times more likely to implement nursing process. Additionally, nurses who attained any course, talk, or seminar related to the nursing process recently on the ward or in the hospital were 15 times and nurses who have been taught about nursing process adequately during studies at the nursing school were 7 times more likely to implement nursing process.

But factors like years of experience, the unit in which nurses working in during data collection time, the number of patients they give care, amount of time nurses working per day, and a monthly salary of nurses were not associated with the implementation of nursing process in our study (Table 3).

### 3.5. Factors Affecting the Implementation of Nursing Processes

83.3% of respondents said that lack of preparedness or knowledge about the nursing process or some part of it is the most common factor that affects implementation of nursing process. An absence of in-service training pertinent to nursing process (75.5%), low satisfaction level (73.5%), low salary (69.6%) and lack of practice in the implementation of nursing diagnosis (61.8%) were also recurrently mentioned factors that affect implementation of a nursing process (Table 4).

## 4. Discussion

Nursing process is the essence and scientific bases of nursing profession. Though the level of implementation varies from country to country, it is being implemented almost throughout the world, and in some countries it is used as a standard of care [6]. In Ethiopia, more than any time before, the process is getting an attention. In the current study, however, among 102 study participants, only 43 (42.1%) nurses were implementing nursing process. Considering standards of care and the Ethiopian hospitals reform implementation guideline recommendations this result was very low. Similarly it was lower than a study conducted in Addis Ababa in which nursing process implementation was about 52.1% [10]. Some of the reasons for this low level of implementation might be lack of knowledge about the process, being inadequately taught about nursing process, lack of in-service training, less experienced nurses, low level of education, uncomfortable weather condition, poor administrative support, etc. Of course, the result was higher than other studies conducted in other regions of Ethiopia; Arbaminch 32.7%, Mekele zone not yet implemented, and Finoteselam and Debremarkos hospitals 37.1% [5, 12, 13].

When the level of implementation by each step was analyzed in this study, assessment and diagnosis were carried out by 57 (56.9%) nurses, planning by 46% of nurses, implementation by 38.2% of nurses, and evaluation by 36.2% of nurses. This finding is lower than the study conducted in Brazil where assessment and evaluation were performed in more than 90% of cases; planning was made in 74.8% of cases. In terms of diagnosis, however, the current study is higher than the study conducted in Brazil in which no diagnosis was performed [7]. Nevertheless, Brazil's study focus was only diagnosis. Therefore variation could be methodological. On the other hand, compared to study conducted in Kenya, the current study is higher in all steps in which the implementation of each step ranged between 15.7% and 30.1% [14]. The variation might be possibly due to sample size difference and methodological variation.

To effectively implement nursing process, one should be knowledgeable about the process and its parts. In the current study, about 83% of respondents said that lack of preparedness or knowledge about the nursing process or some of its parts affects implementation of nursing process. The same is found in different studies where lack of proper knowledge on the concept or on how to implement the process has been the most important factor in preventing a proper implementation of the process [4, 5, 10, 12, 15–17]. The lack of knowledge in current study could be attributed to the way of teaching recruited by teachers and attention given for nursing process. For example, most of the time, teachers use a medical approach in teaching-learning process. If nurses have to implement the process, they should be taught adequately. In this regard, a quasi-experimental study conducted in Iran showed the positive effects of being taught adequately about nursing process on its implementation [18]. The present study also showed that nurses who have been taught about nursing process adequately during studies at the nursing School were 7 times (COR=7.33, 95% CI [2.82-19.08], and p <0.01) more likely to implement nursing process than those who have not been taught adequately. Unfortunately, most (69.6%) of the respondents reported that they were not taught nursing process adequately. Certainly, the problem with governmental school is not curriculum related, as its framework is nursing process. The problem might be the inadequate attention given for nursing process during curriculum implementation. In private institutions, however, the problem could also be curriculum related. While all governmental institutions in Ethiopia use a harmonized curriculum, private institutions use varied curriculum. This could also be one of the reasons why in the present study governmental institution graduate nurses were 2 times more likely to implement nursing process than the private institution graduates. Therefore giving special attention to curriculum harmonization in between governmental and private institution and monitoring and evaluation of its implementation would bring change in implementation of nursing process at the realm of practice.

On the other hand, the level of education determines level of knowledge about nursing process and consequently about its implementation. In the present study, bachelor degree holder nurses were 4 times more likely to implement nursing process than diploma holders. On the other hand, majority (65.7%) of the respondents in this study were diploma holders which had contribution to low level of nursing process implementation. The difference in level of implementation makes it clear that bachelor nurses learn nursing process more than diploma nurses.

Seventy-six (75.5%) of respondents in this study mentioned that absence of training was a reason for not implementing nursing process. Training is a key to keep momentum of proper implementation of nursing process [19, 20]. A study conducted in Egypt identified that 93.3% of respondents believe that receiving practical training in the hospital facilitates execution of nursing process [16]. The current study also showed that nurses, who attained training recently (during data collection time) in the ward or in the hospital, were 15 times more likely to implement the process. While this is the fact, training accessibility is a problematic issue in developing countries. A question was raised as to whether they attended any talks or seminars related to nursing process recently in the ward or in the hospital and 70.6% of this study respondent said no. Lack of training in Afar region is majorly due to its peripherality from the capital city of Ethiopia, as most of trainings took place either in Addis or around Addis.

As to our belief, the weather condition of the region (thought indirectly) had also a negative impact on level of nursing process implementation. Because of the unfavorable hot weather condition, a staff turnover is high in Afar hospitals. And sometimes competent nurses either do not apply for vacant positions or they will not stay for long. This is why fifty four (52.9%) of the current study participant were having 4 and below 4 years of experience.

## 5. Conclusion

Ideally, all nurses in all setting are expected to apply nursing process, which is innovative and problem solving tool. In practice, however, nurses are not implementing the tool yet as expected. In this study nursing process was only partially implemented (42.2%). Lack of knowledge and absence of in-service training were the most common factors hindering its implementation.

## Figures and Tables

**Table 1:** The frequency distribution of the Respondents by their sociodemographic characteristics in Afar region hospitals, Afar, north East Ethiopia, 2016 (n= 102).

Variable		Frequency	Percentage
Sex	Male	54	52.9
	Female	48	47.1
Age	20-24	33	32.4
	25-29	55	53.9
	≥30	14	13.7
Educational status	Diploma	67	65.7
	BSc	35	34.3
Institution from where educational award is obtained	Government	76	74.5
	Private	26	25.5
Year of experience	0-4	54	52.9
	5-9	30	29.4
	≥10	18	17.6
The unit in which the respondents are currently working in	Medical	33	32.4
	Surgical	23	22.5
	Pediatrics	16	15.7
	Ob-gyn	18	17.6
	Others	12	11.8
The amount of hours respondents working per day	≤8 hours	60	58.8
	>8 hours	42	41.2
Numbers of patients to whom respondents give service	<5	20	19.6
	5-10	44	43.1
	11-15	19	18.6
	>15	19	18.6
Monthly salary of respondents (Ethiopian Birr)	1500-2000	42	41.2
	2001-2500	15	14.7
	2501-3000	13	12.7
	=>3000	32	31.4
Attending any talk or seminar related to nursing process recently on the ward or in the Hospital	Yes	30	29.4
	No	72	70.6
Taught about nursing process adequately during studies at the Nursing School	Yes	31	30.4
	No	71	69.6

**Table 2:** Level of implementation of nursing process among nurses in Afar region hospitals, Afar, north East Ethiopia, 2016 (n= 102).

Questions	Responses (N=102)				
	Never	Don't know	Sometimes	Usually	Always
Assessment and diagnosis					
(1) Is an assessment made of new patients prior to planning	2(2%)	1(1%)	12(11.8%)	27(26.5%)	60(58.5%)
(2) Is written nursing history taken using a specific form	7(6.9%)	5(4.9%)	16(15.7%)	41(40.2%)	33(32.4%)
(3) Does the nursing assessment begins within 24 hours	6(5.9%)		15(14.7%)	30(29.4%)	51(50%)
(4) Does the assessment of the patient conclude with identification of problems	8(7.8%)	2(2%)	19(18.6%)	45(44.1%)	28(27.5%)
(5) Is an attempt made to find the cause of the problems	8(7.8%)	3(2.9%)	25(24.5%)	36(35.3%)	30(29.4%)
(6) Are problem statements arranges in order of priority	3(2.9%)	6(5.9%)	27(26.5%)	47(46.1%)	19(18.6%)
(7) Are patients'/or relatives opinions systematically takes in to account	5(4.9%)	7(6.9%)	33(32.4%)	42(41.2%)	15(14.7%)
Planning					
(1) Is a written care plan made before carrying out nursing interventions on the patient?	6 (5.9%)	7(6.9%)	22(21.6%)	36(35.3%)	31(30.4%)
(2) Does the nursing care plan incorporate the nursing problems identified?	5(4.9%)	2(2%)	30(29.4%)	36(35.3%)	29(28.4%)
(3) Are goals for the resolution of each one of the problems identified and documented in the care plan?	4(3.9%)	5(4.9%)	26(25.5%)	41(40.2%)	26(25.5%)
(4) Do the goals provide enough detail (i. e. time to be accomplished, who will accomplish what and how?	4(3.9%)	5(4.9%)	33(32.4%)	42(41.2%)	18(17.6%)
(5) Are nursing interventions identified and documented in the care plan?	5(4.9%)	5(4.9%)	19(18.6%)	41(40.2%)	32(31.4%)
(6) Are planned nursing interventions written with enough detail?	6(5.9%)	8(7.8%)	23(22.5%)	47(46.5%)	18(17.6%)
(7) Is there any systematization on the ward to take into account patient/relatives opinions regarding the goals and planned activities?	10(9.8%)	4(3.9%)	32(31.4%)	39(38.2%)	17(16.7%)
(8) Are nursing care planning discussions held on the ward?	17(16.7%)	6(5.9%)	25(24.5%)	34(33.3%)	20(19.6%)
(9) Do you periodically read professional journals or do you take part in research projects in order to update your practice accordingly?	5(4.9%)	8(7.8%)	26(25.5%)	46(45.1%)	17(16.7%)
Implementation					
(1) Is the patient's condition reassessed before implementing any planned nursing intervention in order to be sure of its appropriateness?	9(8.8%)	7(6.9%)	26(25.5%)	47(46.1%)	13(12.7%)
(2) Are nursing interventions explained to patients and/or relatives and their opinions taken into account?	8(7.8%)	8(7.8%)	29(28.4%)	42(41.2%)	15(14.7%)
(3) Is the way patient and /relatives should participate in their care systematized?	3(2.9%)	6(5.9%)	39(38.2%)	42(41.2%)	12(11.8%)
(4) Is patient allocation or primary nursing used throughout the ward all times?	6(5.9%)	6(5.9%)	39(38.2%)	38(37.3%)	13(12.7%)
(5) Are nurses allocated to the same patients for several days?	13(12.7%)	6(5.9%)	38(37.3%)	35(34.3%)	10(9.8%)
(6) Do nurses take part in medical round for their patients?	3(2.9%)	4(3.9%)	24(23.5%)	49(48%)	22(21.6%)
(7) Are nurses in this hospital responsible for the planning of patient care? That is, the nurse and not the supervisor.	3(2.9%)	4(3.9%)	28(27.5%)	48(47.1%)	19(18.6%)
(8) Is it compulsory on this ward to work with the nursing process approach?	13(12.7%)	4(3.9%)	34(33.3%)	34(33.3%)	17(16.7%)
(9) Is nursing documentation kept once the patient has been discharged?	2(2%)	4(3.9%)	29(28.4%)	38(37.3%)	29(28.4%)
(10) Are care plans used both day and night as a basis for giving care?	3(2.9%)	3(2.9%)	35(34.3%)	41(40.2%)	20(19.6%)
Evaluation					
(1) Is a systematic evaluation made of the effectiveness of care given to solve patient nursing problems?”	3(2.9%)	5(4.9%)	37(36.3%)	37(36.3%)	20(19.6%)
(2) Is the evaluation recorded in the care plans or progress notes?	3(2.9%)	6(5.9%)	37(36.3%)	41(40.2%)	15(14.7%)
(3) Are objective measures of patient progress towards the identified goals used on the ward?”	3(2.9%)	6(5.9%)	35(34.3%)	44(43.1%)	14(13.7%)
(4) Are care plans modified according to the results of evaluation? (example: to add new ones, stop others, )	5(4.9%)	2(2%)	43(42.2%)	40(39.2%)	12(11.8%)
(5) Is there a systematic way in which patient/relatives participate in the evaluation of care on the ward?	13(12.7%)	6(5.9%)	39(38.2%)	35(34.3%)	9(8.8%)

Source: Amparo Zaragora Salcelo (2004) @ http://theses.gla.ac.uk/3718/.

**Table 3:** Binary logistic analysis and association of implementation of nursing process by selected variable among nurses in Afar region Hospitals, north East Ethiopia, 2016 (n= 102).

Variable	Implementation of nursing process		P-value	COR(CI)
	Yes	No		
Sex				
Male	28(51.9%)	26(48.1%)	0.037	2.369(1.053, 5.330)∗
Female	15(31.2%)	33(68.8%)		1
Age of respondents				
20=24	10(30.3%)	23(69.7%)		1
25-29	24(43.6%)	31(56.4%)	0.174	2.325(0.689, 7.845)
≥30	9(64.3%)	5(35.7%)	0.035	4.14(1.104, 15.521) ∗
Educational level of respondents				
Diploma	20(29.9%)	47(70.1%)		1
BSC	23(65.7%)	12(34.3%)	0.002	4.194(1.731, 10.164) ∗∗
Institution from where educational award is obtained				
Government	38(50%)	38(50%)	0.009	4.2(1.435, 12.293) ∗∗
Private	5(19.2%)	21(80.8%)		1
Year of experience				
0-4	16(29.6%)	38(70.4%)		1
5-9	17(56.7%)	13(43.3%)	0.940	0.956(0.295, 3.102)
≥10	10(55.6%)	8(44.4%)	0.052	2.969(0.990, 8.900)
The unit where nurses work in the hospital				
Medical	16(48.5%)	17(51.5%)	0.096	2.667(0.841, 8.458)
Surgical	6(26.1%)	17(73.9%)	0.756	1.21(0.364, 4.02)
Pediatrics	7(43.8%)	9(56.2%)	0.511	1.479(0.46, 4.755)
Obstetrics-gynecology	7(38.9%)	11(61.1%)	0.56	0.672(0.177, 2.555)
Others	7(58.3%)	5(41.7%)		1
Working hours per day				
≤8 hours	21(35.0%)	39(65.0%)	0.082	0.490(0.219, 1.095)
≥8 hours	22(52.4%)	20(47.6%)		1
Number of patients respondents give care at a time				
<5	9(45.0%)	11(55.0%)	0.839	0.896(0.310, 2.589)
5-10	21(47.7%)	23(52.3%)	0.882	0.909(0.258, 3.204)
11-15	9(47.4%)	10(52.6%)	0.120	3.068(0.748, 12.587)
>15	4(21.1%)	15(78.9%)		1
Monthly salary of respondents				
1500-2000	13(31%)	29(69%)		1
2001-2500	7(46.7%)	8(53.3%)	0.831	1.143(0.335, 3.904)
2501-3000	7(53.8%)	6(46.2%)	0.099	2.231(0.860, 5.785)
>3000	16(50%)	16(50%)	0.815	0.857(0.235, 3.120)
Attending any course, talk or seminar related to the NP recently on the ward or in the Hospital				
Yes	25 (83.3%)	5 (16.7%)	≤0.001	15(5.001-44.991) ∗∗
No	18 (25%)	54 (75%)		1
Taught about nursing process adequately during studies at the Nursing School				
Yes	23 (74.2%)	8(25.8%)	≤0.001	7.33(2.82-19.08) ∗∗
No	20(28.2%)	51(71.8%)		1

Note ∗ means association is significant at p≤0.05 and ∗∗ means association is significant at p≤0.01

**Table 4:** The frequency distribution of the respondents opinion regarding the factors affecting implementation of nursing process in Afar region Hospitals, north East Ethiopia, 2016 (n= 102).

Question	Response (n=102)					
	Yes		No		I don't know	
	F	%	F	%	F	%
Lack of preparedness or knowledge about the nursing process or some part of it	85	83.3	16	15.7	1	1
Absence of in-service training pertinent to nursing process	77	75.5	24	23.5	1	1
Lack of practice in implementation of nursing diagnosis	63	61.8	38	37.3	1	1
Lack of time	50	49	51	50	1	1
High patient flow	48	47.1	53	52	1	1
High patient to nurse ratio	53	52	49	48	--	--
Early patient discharge	35	34.3	55	53.9	12	11.8
Lack of resource	43	42.2	56	54.9	3	2.9
Lack of supports from colleagues	43	42.2	58	56.9	1	1
Poor/no support from administrators	57	55.9	44	43.1	1	1
Lack of monitoring and evaluation	57	55.9	44	43.1	1	1
Lack of motivation for implementers	60	58.8	41	40.2	1	1
Low satisfaction level	75	73.5	27	26.5	--	--
Low salary	71	69.6	31	30.4	--	--
Poor patient economic status	36	35.3	65	63.7	1	1
Patients awareness towards nursing process	35	34.3	62	60.8	5	4.9
Uncooperative patient	33	32.4	65	63.7	4	3.9

## Data Availability

The data used to support the findings of this study are available from the corresponding author upon request.
